# Airway Pressure Release Ventilation With Time-Controlled Adaptive Ventilation (TCAV™) in COVID-19: A Community Hospital’s Experience

**DOI:** 10.3389/fphys.2022.787231

**Published:** 2022-04-05

**Authors:** Philippe Rola, Benjamin Daxon

**Affiliations:** ^1^Intensive Care Unit, Santa Cabrini Hospital, Montreal, QC, Canada; ^2^Mayo Clinic, Rochester, MN, United States

**Keywords:** ARDS, COVID-19, APRV, TCAV, respiratory failure, critical care

## Abstract

Santa Cabrini Ospedale, a community hospital in Montreal, Canada, used the airway pressure release ventilation following a time-controlled adaptive ventilation (APRV-TCAV™) approach for several patients in the first wave of the coronavirus disease 2019 (COVID-19) outbreak in the spring of 2021. Based on favorable patient responses, it became the primary mode of invasive mechanical ventilation—from initiation through extubation—during the second and third waves of COVID-19. In this article, we describe our success with APRV-TCAV™ over more conventional modes and protocols and look at three cases that aptly demonstrate our experience. We then outline several risks with our approach and the lessons learned from our experience. While we generally saw improvement in patients’ clinical course with APRV-TCAV™, there are inherent risks with this approach that others must prepare for if they attempt to implement it in their practice.

## Introduction

The coronavirus disease 2019 (COVID-19) pandemic hit the world in January of 2020 and reached Montreal, Canada, in March of that year. The harrowing reports of COVID-19’s proclivity to develop acute respiratory failure with exceedingly high mortality were concerning to our critical care team. Of particular concern was the atypical nature of the COVID-19-associated acute respiratory distress syndrome (C-ARDS). Many patients displayed profound hypoxia with relatively little dyspnea—“happy hypoxics”—raising numerous questions about pathophysiology and treatment implications (Ferguson et al., 2020; Goligher et al., 2021). There were also early reports of dichotomized L and H phenotypes, which perhaps necessitated differing ventilatory strategies (Gattinoni et al., 2020; Marini and Gattinoni, 2020). While the optimum ventilator strategy for C-ARDS was unclear, a low tidal volume (LTV) or Acute Respiratory Distress Syndrome Network (ARDSnet) strategy was endorsed by most guidelines and widely applied (Alhazzani et al., 2020; Alqahtani et al., 2020; ANZICS, 2020). But the rapid multiorgan failure following intubation and high mortality rates belied confidence that this ventilatory approach was best suited to C-ARDS. The LTV approach utilizes a tidal volume based on the ideal body weight, but lung volume correlates poorly with this; furthermore, the pathologies causing C-ARDS do not affect the lung parenchyma according to a weight-based pattern.

One of the silver linings of the COVID-19 pandemic was the increased level of collaboration that occurred among medical professionals across the globe. While the online medical community has been gaining momentum in recent years through blogs, podcasts, Twitter, etc., under the rubric of Free Open-Access “Meducation” (FOAMed), the pandemic greatly accelerated this community’s growth and impact. Through this collaboration, our critical care team at Santa Cabrini Ospedale explored the literature around airway pressure release ventilation (APRV) and posited that APRV following a time-controlled adaptive ventilation (TCAV) strategy (i.e., APRV-TCAV™) might provide ventilation better tailored to the diverse and unique pathologies of C-ARDS. And since our center did not have extracorporeal membrane oxygenation (ECMO) capabilities, we thought it all the more important to utilize a ventilatory strategy that could adequately support oxygenation.

Our hospital trialed the mode on select patients during the first wave and, in certain cases, achieved remarkable success. Given this success, we then transitioned to utilizing APRV-TCAV™ as the primary mode for all intubated COVID-19 patients during the second and third waves and found our care markedly improved from what was expected using more traditional LTV approaches. This success came about through trial and error, however, and many lessons were learned that now inform a more thoughtful, safe, and effective way to use the mode and protocol.

## The Airway Pressure Release Ventilation With Time-Controlled Adaptive Ventilation™ Approach: A Cursory Review

In brief, APRV is a mode of ventilation that alternates between two levels of continuous positive airway pressure (CPAP) and allows for spontaneous breathing throughout the entirety of the respiratory cycle. It requires setting four parameters, namely, the pressure at each level of CPAP and the time spent at each—pressure high (P_High_), pressure low (P_Low_), time high (T_High_), and time low (T_Low_). These parameters are guided by a protocol referred to as time controlled adaptive ventilation (TCAV). TCAV™ starts by setting the P_High_ at the prior mode’s plateau pressure for a T_High_ that is based on the prior respiratory rate. A faster respiratory rate will result in a shorter T_High_ and a slower rate will result in a longer T_High_. In general, most patients with severe ARDS require a P_High_ of 25–30 cm H_2_O, and a T_High_ of 2.5–6 s. This time at a high level of CPAP is punctuated by infrequent, brief drops in pressure to zero, P_Low_, and referred to as a “release.” The duration of the releases, T_Low_, is usually very short and depends upon the expiratory flow curves as described below. Put as succinctly as possible, TCAV™ is a high CPAP with brief releases.

The extended time at a higher CPAP results in markedly increased mean airway pressures without concomitant increases in peak or plateau pressures. Over time, a lung subjected to these higher mean airway pressures is able to recruit and “open” (assuming the lung has not entered a fibroproliferative phase and all other clinical parameters being equal). The functional residual capacity can be regained, and more normal, homogenous lung architecture can be restored. In fact, APRV is sometimes described as simply a prolonged and judicious recruitment maneuver.

**FIGURE 1:**
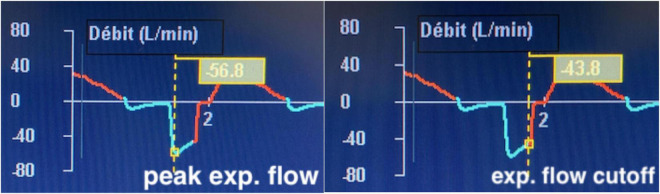
Manual adjustment of TLow: peak expiratory flow is measured (left) and the TLow is set to obtain a cutoff at or above 75% of peak flow, in this case 77%.

**FIGURE 2:**
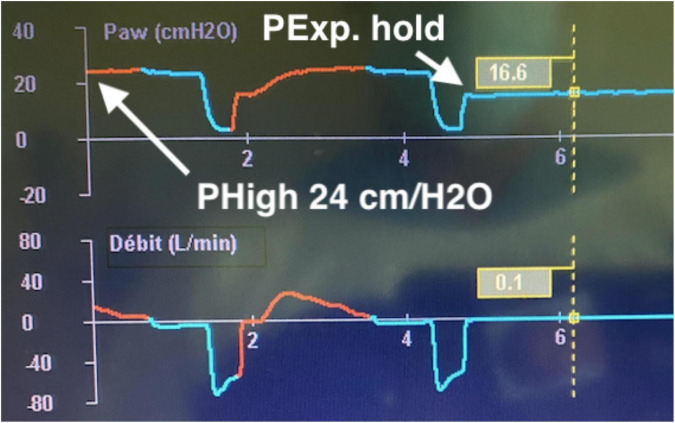
Calculation of Effective PEEP/Driving pressure: an expiratory hold is performed and equilibration of pressure occurs. This is the effective PEEP. Driving pressure is PHigh – PExpiratory hold, in this case approximately 7–8 cmH2O.

To maintain sufficient minute ventilation and to offload some of the ventilatory burden on the patient, releases are employed. While the release pressure, P_Low_, is set to zero, some level of intrinsic positive end-expiratory pressure (iPEEP) is retained by terminating the release breath while there is still significant expiratory flow. This is performed by measuring the peak expiratory flow and adjusting the T_Low_ such that it cuts off expiration at or above 75% of peak flow (Figure 1). In some ventilators, it is carried out automatically, and once the expiratory flow decays by 25%, the T_Low_ terminates and pressure reverts back to P_High_. In other words, the T_Low_ is set according to the time it takes the expiratory flow to decay to 75% of the peak expiratory flow. “Trapping” of air based on a time is the key element of this ventilatory strategy and results in an auto-peep that stabilizes the lung by minimizing the alveolar size variation during expiration. Initial *in vivo* animal experiments showed that with a cutoff of 75%, most of the exhaled air was from the conducting airways with a minimal change in the alveolar size (Nieman et al., 2018; Kollisch-Singule et al., 2019) thereby “splinting” the alveoli open. This air splint, in theory, minimizes atelectrauma and maximizes the maintenance of recruitment. While many misinterpret the settings as indicative of a driving pressure of P_High_—P_*Low*_ (which would be excessively high in most cases) the driving pressure to the alveoli is in fact P_High_—intrinsic PEEP. This intrinsic PEEP is easily confirmed with a brief expiratory hold, which always demonstrates a marked discrepancy between the set P_Low_ of zero and the measured airway pressure during a hold (Figure 2). Setting the P_Low_ and T_Low_ in this manner has been demonstrated in numerous animal studies to keep the alveoli stable, i.e., they do not collapse with each breath (Nieman et al., 2018).

As the lung recruits, the alveoli get a chance to heal, and if the patient’s overall condition improves, the higher pressures can be gradually reduced and the number of releases spread out, a process referred to as “drop and stretch.” With this, the patient takes on a larger share of the ventilatory burden, and support is slowly withdrawn until the patient reaches a point of breathing almost entirely on a lower level of CPAP.

Several points bear highlighting. First, the entirety of ventilation can be accounted for by the releases so that APRV can be used on paralyzed patients. In this case, any benefit from spontaneous breathing will be lost, but given the potential risks of patient self-inflicted lung injury (P-SILI), this may not necessarily be a bad thing. Second, nothing prevents APRV-TCAV from being used in a prone position. Third, restoration of lung volume and improvement in V/Q matching often result in improved oxygenation, an “apparent cure,” before the previously injured alveoli have had a chance to heal. As a result, many clinicians often prematurely reduce the pressures, and as a result, alveoli quickly collapse again. Finally, tidal volume and traditional PEEP are not directly set. While P_High_, P_Low_, T_High_, and T_Low_ can be adjusted to account for preferred tidal volumes and PEEP levels, their lack of direct control often results in clinicians adjusting the mode in ways that might be deleterious in the context of TCAV™, e.g., extending the T_Low_ to achieve more minute ventilation. Finally, not all APRV is TCAV™. While many practitioners claim to practice a TCAV™ approach, the settings outlined above are precise and interlinked. Deviation from them in even small ways can result in profound harm. For a more extensive discourse on the mode and protocol, we refer readers to Kollisch-Singule et al. (2019) and Habashi et al. (2021).

## Overview of Our Experience Through the Different Waves

As one of the designated centers for COVID-19, our center was rapidly overwhelmed with deteriorating patients. Profound hypoxia was the rule, and we liberally used non-invasive ventilation and awake proning on the wards. Despite this, many deteriorated further, and our expanded-26-bed intensive care unit (ICU) had over 90% of its patients intubated, many requiring frequent proning cycles.

In the first wave, we saw a preponderance of elderly patients and an overall mortality rate of 67%, which is consistent with reports in the literature (Hyman et al., 2020; Luo et al., 2020). APRV-TCAV™ was mostly used as a rescue therapy since the medical and respiratory therapy teams were not familiar or comfortable with the APRV-TCAV™ approach. Despite this lack of experience, APRV-TCAV™ still rescued a few seemingly intractable cases, and we decided to leave those patients on the mode for the remainder of their course. They were among the few survivors of the ventilated cohort. While the numbers are too small to draw meaningful conclusions, they piqued our interest and established the mode as a viable alternative.

After reviewing our first-wave experience, delving into the literature, and discussing with colleagues at other institutions, we proceeded with APRV-TCAV™ as the primary mode of ventilation from intubation to liberation for the majority of cases during the second and third waves. Refractory hypoxia was limited to a few cases unresponsive to TCAV™, which usually ended up requiring transfer for ECMO. With a decreased incidence of refractory hypoxemia came a concurrent decrease in proning—a welcome relief from the nursing work intensity that was needed on other modes of ventilation during the first wave.

In most cases, the following would occur in, approximately, the first 24 h:

(a) Oxygen requirements would significantly decrease.
(b) Driving pressures would markedly decrease, often to less than 10 cm/20 h.
(c) Chest imaging would improve substantially.

All this would occur without substantial deterioration in other organ functions, e.g., there was no escalation of vasopressors, need to initiate continuous renal replacement therapy (CRRT), or worsening sedation needs. If anything, the patients’ overall condition would improve in concert with improvement on the ventilator.

## Challenges and Considerations When Using Airway Pressure Release Ventilation With Time-Controlled Adaptive Ventilation™

### False Reassurance by Improved Oxygenation

These improvements were often misleading, however. Several consultants unfamiliar with APRV-TCAV™ were surprised that the patients were not being more aggressively weaned since they “clearly no longer had ARDS” based on their FiO_2_ and chest X-ray (CXR). But while the lung may have been recruited and traditional parameters were improved, there was still an underlying pathology that required additional time to heal. If the P_High_ was decreased before the true alveolar stability was achieved, then the CXR would abruptly worsen and gas exchange would quickly regress. This phenomenon of needing to “stabilize” the lung after recruitment on APRV-TCAV™ has been reported elsewhere and likened to needing a cast for a period of time after setting a fracture (Nieman et al., 2018). Hence, clinicians will have to carefully observe patients’ breathing patterns, ventilator curves, and gas exchange during weaning.

### Risks With Changing Modes

The clinical improvement associated with the use of TCAV™ must be taken into account when considering a change of modes. While changing from a traditional pressure-control mode to an assist-control mode (given similar tidal volumes and PEEP) would not likely result in large swings in oxygenation, this does not always hold true for TCAV™. The clinician should clearly understand that there are several elements that will impact oxygenation in a potentially drastic way, especially if the change occurs before the alveolar stability is achieved as described above. First, TCAV™ is similar to the inverse ratio in that more time is spent at a higher pressure. Hence, switching to alternative modes with roughly the same high and low pressures but a substantially different time at those pressures can result in a significant drop in mean airway pressure. Second, if alveolar stability is not achieved, patients will likely derecruit. The rapidity and severity of deterioration are difficult to predict but can occur in minutes and likely correlate to the required P_High_ and the time spent there previously.

In our experience, these premature and/or inappropriate transitions occurred most often after hand-offs between treating physicians and during patient transport. When a physician is unfamiliar with TCAV™ and the clinical state requires a change to the settings, the clinician often changes to a mode that is more common and familiar. Transitioning to an alternative mode from APRV-TCAV™ before a lung has stabilized is fraught with complexities and nuance that is usually more difficult than making an adjustment to the APRV-TCAV™ settings. It is in this back-and-forth where patients are at most risk for harm and is one of the main reasons why a team buy-in is necessary. This holds true for patient transport as well where portable ventilators are often incapable of providing APRV-TCAV™. Hence, when deciding on, say, a CT scan, the risks of transporting with an alternative ventilatory mode must be weighed against the benefits of the scan. In our center, bedside ultrasound is used extensively, yet trips to CT scan were still required from time to time. This issue also applies to interhospital transfers.

One potential solution for transfers is to approximate TCAV™ by using inverse ratio ventilation with pressure control. The inspiratory pressure should match the P_High_, and the PEEP should be zero. The inspiratory/expiratory (I:E) ratio will need to reflect the T_High_:T_Low_, often being set at the maximum ratio, and patients may need additional sedation and/or paralysis. APRV utilizes floating valves that allow patients to breathe *ad lib* as they would on CPAP. This is not possible on inverse ratio pressure control and will likely be too uncomfortable for spontaneously breathing patients. Utilizing this approach can often create a respiratory rate around 25 with a T_Low_ equivalent of 0.4 and may come close to matching the termination of expiratory flow at 75% (60/25 = 2.4 s cycle. At an I:E of 5:1 that is a “T_*High*_” of 2 s and a “T_*Low*_” of 0.4 s).

### Bagging the Time-Controlled Adaptive Ventilation Patient

Personnel must understand that bagging is unlikely to help the hypoxic patient on TCAV™ and 100% FiO_2_. Although a time-honored practice, hand-bagging will likely result in a much lower mean airway pressure—even when a PEEP valve is used—and may result in deterioration. Rapidly passing a suction catheter to rule out occlusion, analyzing the ventilator waveform and end-tidal CO_2_ (ETCO_2_), carefully observing the patient, and quickly scanning with point-of-care ultrasound (POCUS) or obtaining a CXR to rule out pneumothorax should be the initial reflexes. Bagging should only be considered after serious deliberation with the clear understanding that this may result in massive derecruitment.

### Timing of Interhospital Transfer

In the escalation of ventilatory therapy, APRV usually sits just below veno-venous ECMO (VV-ECMO). In some cases, it may avoid the need for such escalation (Lim et al., 2016); however, when it is being used in a center that does not have VV-ECMO in-house, it is important for the treating team to realize that the inherent risks of transfers will be heightened if the patient is on maximal support with 100% FiO_2_ and very high APRV-TCAV™ settings. Hence, the transfer should be considered when there is still some margin of safety as transferring may require coming off TCAV, which carries risks as described earlier. For those transfers we carried out, we kept the ventilator on for as much of the physical transfer to the ambulance stretcher and only switched to the transport ventilator when the whole team was ready to roll off with the patient.

## Clinical Vignettes

In the following cases, we have illustrated some of these issues as well as showed the different ways in which APRV-TCAV™ was used.

### Case 1—First Wave

**FIGURE 3:**
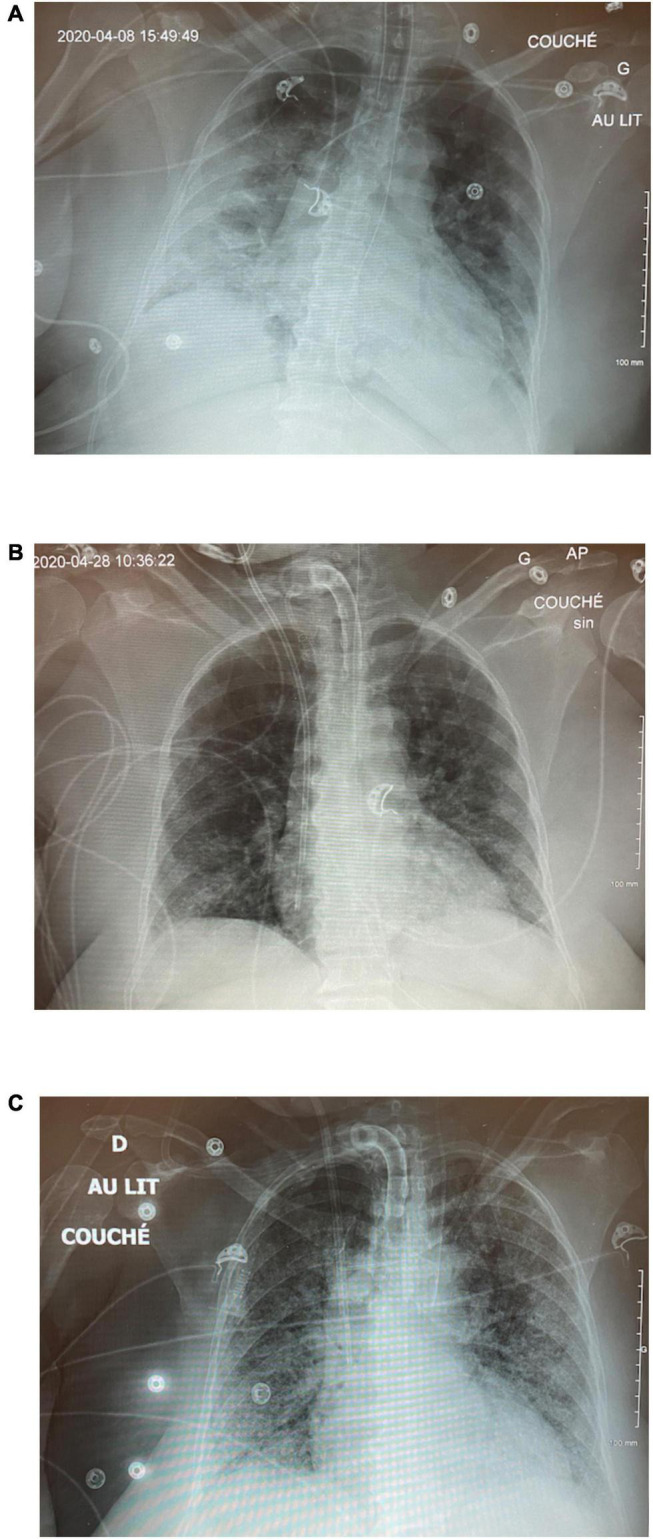
Case 1: **(A)** CXR on ICU day 2 on PCV. **(B)** CXR on day 17 post-tracheostomy. **(C)** CXR following switch to CMV.

A 55-year-old woman was admitted with severe COVID pneumonia initially managed with non-invasive ventilation but, on day 2, was intubated following severe desaturation to 21% SpO_2_. She was initially put on pressure-controlled ventilation (PCV) with an inspired pressure (P_*insp*_) of 24 cm H_2_O, PEEP of 12 cm H_2_O, and an FiO_2_ of 80% (Figure 3A). On the ensuing days, there was little improvement, and she required serial proning. She continued needing an FiO_2_ over 80% and her PCV settings increased to P_*insp*_ of 29 cm H_2_O and PEEP of 15 cm H_2_O with a driving pressure of 19 cm H_2_O. She developed acute kidney injury (AKI) requiring renal replacement therapy. On day 9, PCV was set to inverse ratio ventilation of 2.7:1 with a slight improvement in oxygenation. Her oxygen requirements progressively worsened, and on day 14, while proned and paralyzed on CMV with a plateau pressure of 38 cm H_2_O and FiO_2_ 100% for a PaO_2_/FiO_2_ (P/F) ratio of 58, APRV-TCAV™ was initiated at 34/2.5/0/0.4 (all APRV settings are referred to as P_High_/T_High_/P_Low_/T_Low_). In the next morning, her FiO_2_ was down to 70% on 36/2.3/0/0.4 and later that day was further decreased to 55% with a P/F ratio of 100. Paralytics were discontinued. Over the next 5 days, her P/F ratio rose to 156, FiO_2_ dropped to 45%, and a percutaneous tracheostomy was carried out on day 17 without complication (Figure 3B). There was no hemodynamic instability, and renal replacement therapy was ongoing. Following a changeover of the medical team, on day 23, while on APRV-TCAV with FiO_2_ 30%, a decision was made to switch to CMV. In the next few hours, her oxygen requirements rose progressively with concomitant hemodynamic instability requiring reinstitution of vasopressors. On the next day, with a P/F ratio of 83 on a PEEP of 10 cm H_2_O, she would intermittently desaturate to 30–35% SpO_2_. She was proned, but her SpO_2_ remained in the 60’s with a P/F ratio of 42. At this point, TCAV was reinstated at 42/2.4/0/0.3 in hopes to rerecruit the lungs. In the next 3 h, her SpO_2_ climbed to 92%; however, her P/F ratios never rose above 100 thereafter, and she died suddenly on day 28, without any evidence of pneumothorax.

This case tragically illustrates the dangers of switching modes from APRV-TCAV™ without ensuring that alveoli have had sufficient time to heal and stabilize. It also demonstrates the need for teamwork and buy-in when using a mode with which all team members may not be comfortable. It also shows how excellent gas exchange can occur due to TCAV™ prior to achieving inherent alveolar stability.

### Case 2—Second Wave

**FIGURE 4:**
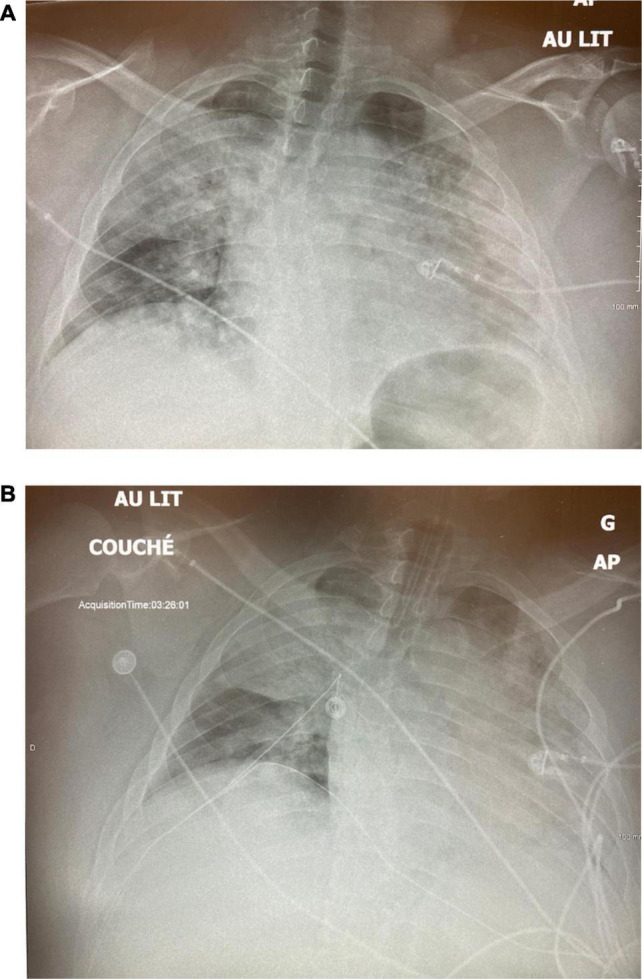
Case 2: **(A)** Day 1 pre-intubation. **(B)** Day 1, 2 h post-intubation, on CMV.

**FIGURE 5:**
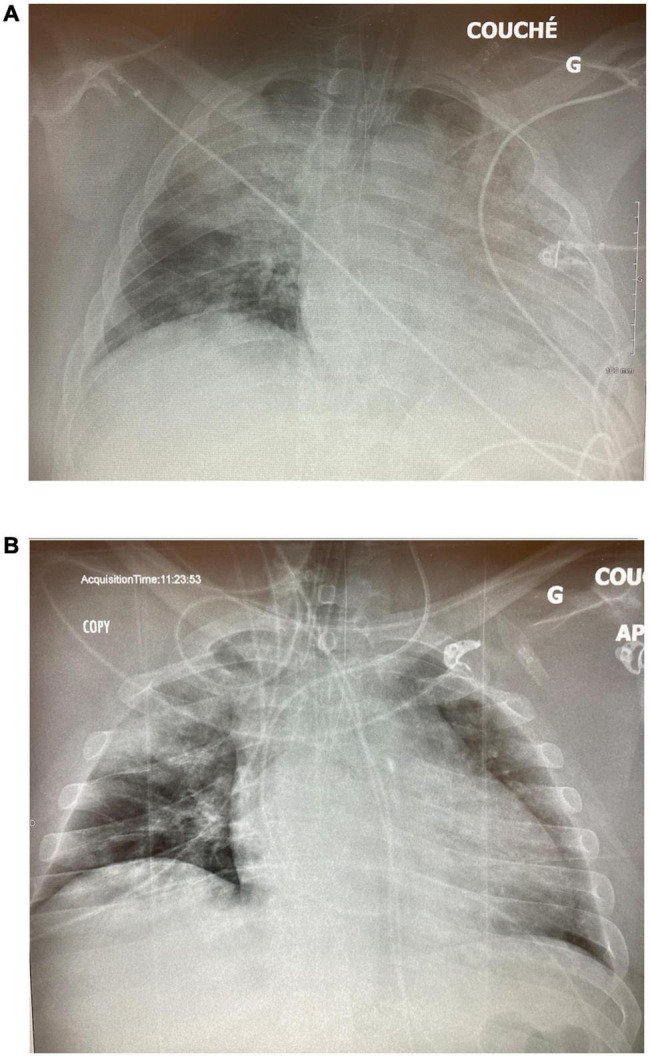
Case 2: **(A)** Day 1, 1 h post APRV-TCAV. **(B)** Day 2, approximately 30 h after initiaition of TCAV(tm).

A 32-year-old man presented to the ED with dyspnea and an SpO_2_ of 60%. He was morbidly obese (150 kg) but with no other past medical history. His CXR showed severe volume loss and bilateral infiltrates (Figure 4). Oxygenation only improved to 84% with high-flow nasal cannula (HFNC) and proning, and the decision was made to intubate him after he displayed respiratory fatigue. Following intubation, the patient suffered from profound desaturation to 20% with severe bradycardia to 18 bpm necessitating atropine, epinephrine, and 2 min of chest compressions. He was placed on CMV, and upon arrival of the critical care team, saturations had increased to 92% on FiO_2_ of 100% and a PEEP of 14 cm H_2_O, as well as an infusion of norepinephrine. With tidal volumes of 400 ml (approx 5 ml/kg ideal body weight), he had a driving pressure of 18 cm H_2_O. His initial arterial blood gas (ABG) showed a pH of 7.28, a PCO_2_ of 59 mmHg, and a PO_2_ of 63 mmHg for a P/F ratio of 63. The CXR 2 hours post-intubation showed almost complete opacification of the left lung and significant infiltrate in the right upper lobe (Figure 4B). He was then put on APRV-TCAV™ 35/1.8/0/0.3 and admitted to the ICU. Approximately, 1 hour after initiation of TCAV™, a second CXR showed slight recruitment of the lower left lobe with some appearance of the diaphragm (Figure 5A). To further augment recruitment, the P_High_ was increased by 2 cm H_2_O to 37 cm H_2_O. Mild hemodynamic instability persisted requiring continued norepinephrine infusion. POCUS revealed a moderately dilated right ventricle, relatively preserved left ventricular function with an ejection fraction of 50%, and a type 2 right ventricular outflow tract (RVOT) pattern indicative of mild pulmonary hypertension (López-Candales and Edelman, 2012). A pulmonary artery (PA) catheter was then inserted to monitor PA pressures, which were 35/25 cm H_2_O at that time.

**FIGURE 6:**
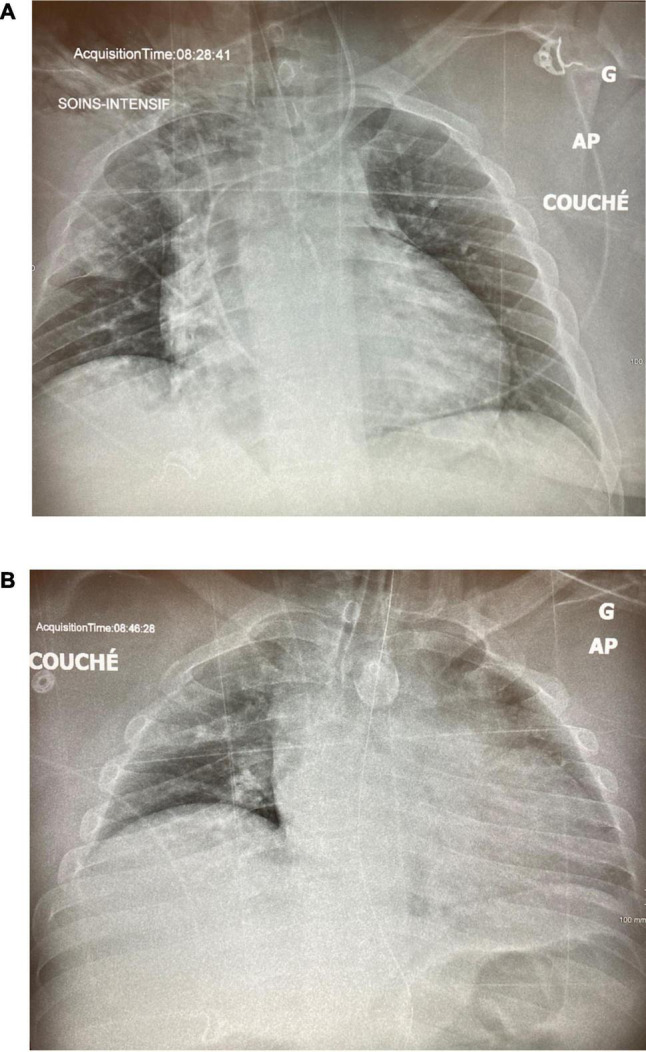
Case 2: **(A)** ICU Day 5. **(B)** ICU Day 13.

About 14 hours following TCAV™ initiation, his FiO_2_ was decreased from 100 to 35% and the P/F ratio rose to 300, while the driving pressure decreased from 18 to 11 cm H_2_O. By the next morning, his CXR showed significant recruitment of the left lung (Figure 5B), and norepinephrine was weaned off. He developed some AKI, but given adequate cardiac output, moderately elevated CVP, and grade 1 venous excess ultrasound (VExUS) score, fluids were not deemed beneficial (Beaubien-Souligny et al., 2020). On day 5, the driving pressure had further decreased to 6 cm H_2_O and the CXR showed further recruitment (Figure 6A). A small, probable pneumopericardium was noted and followed carefully but never required intervention. Over the next few days, his FiO_2_ remained between 25 and 35% with driving pressures below 10 cm H_2_O. His sedation was lightened, he was allowed to breathe more, and his AKI resolved, but his P_High_ could not be decreased below 33 cm H_2_O without desaturation. On day 13, he was dropped to a P_High_ of 28 cm H_2_O, but there was a clear loss of volume/derecruitment on CXR (Figure 6B) over 24 hours, despite PaO_2_ remaining reasonable at 67 mmhg on 25% FiO_2_. At this point, the P_High_ was increased to 33 cm H_2_O as it was felt that alveolar stability had not been reached. Over the next few days, he remained stable and eventually tolerated dropping the P_High_, and he finally was extubated on ICU day 18. This patient never required paralysis, nor proning, partly because of his body habitus, but also because once recruited, his P/F ratios never required it.

This case illustrates the concept of alveolar stability, which is not typically part of the daily assessment of most critically ill ventilated patients but is an inherent part of the TCAV™ approach. It is likely that had the patient been rapidly weaned and extubated after his initial improvement in oxygenation, he would have required reintubation and regressed to a condition worse than after his initial intubation. His reassuring CXRs and persistent P/F ratios >200 nicely demonstrate the concept of “apparent cure” before the alveolar stability is achieved. It should also be noted that the patient had a profound drop in driving pressure, 18 to 6 cm H_2_O, suggesting a significantly reduced level of mechanical power or energy delivered to the lung parenchyma (Costa et al., 2021).

### Case 3—Third Wave

**FIGURE 7:**
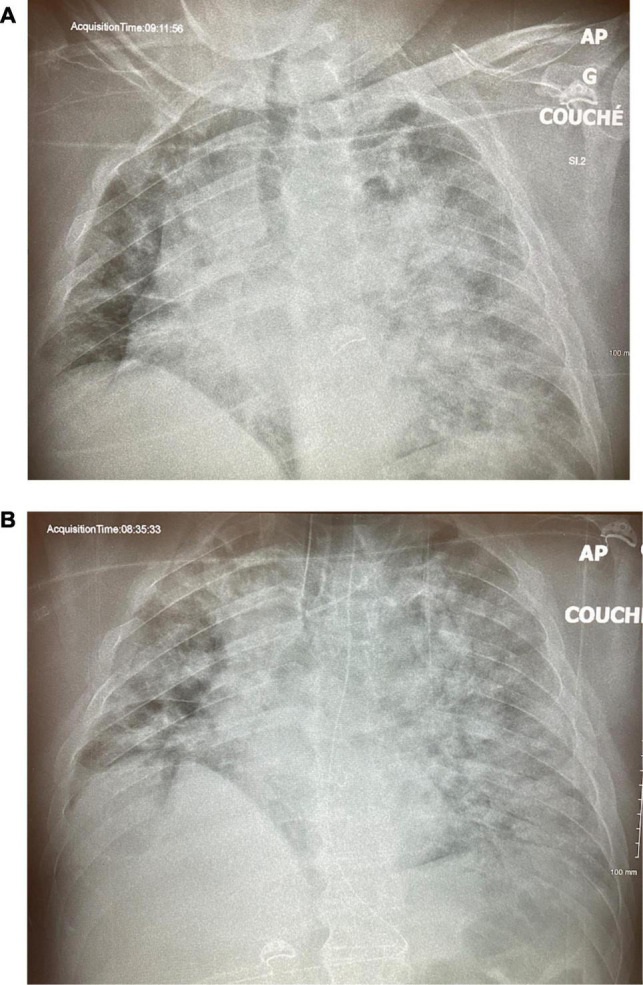
Case 3: **(A)** CXR on Day 1 of admission to ICU, pre-intubation. **(B)** CXR on ICU Day 2, after 20 h of ventilation on PEEP 16cm/H_2_O.

**FIGURE 8:**
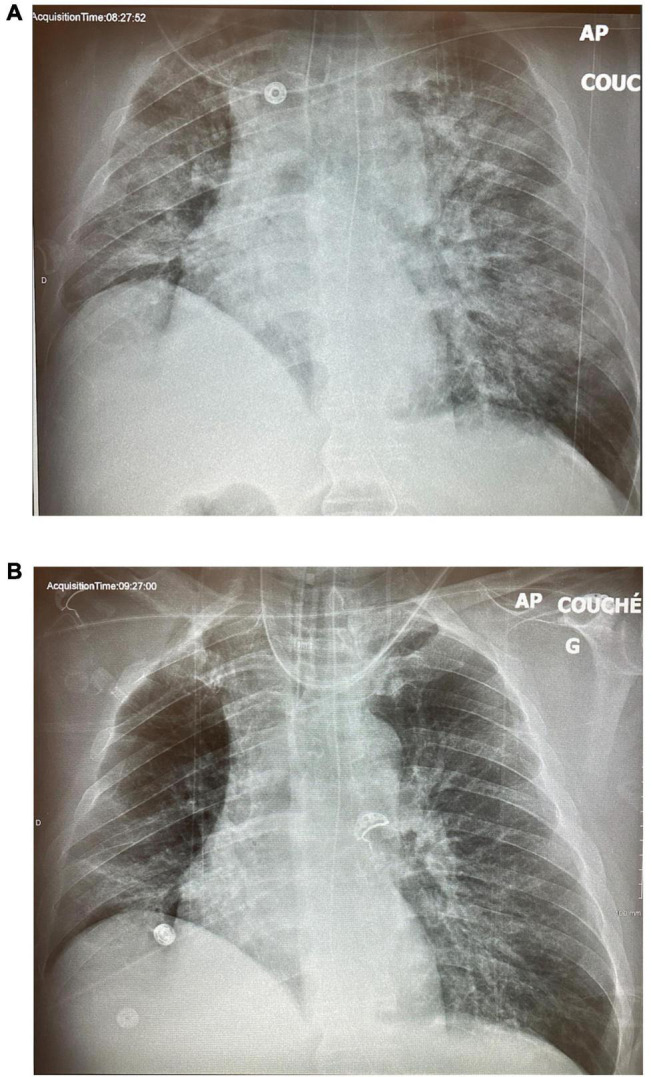
Case 3: **(A)** CXR on ICU Day 3, after 24 h of APRV-TCAV(tm). **(B)** CXR on ICU Day 6.

**FIGURE 9:**
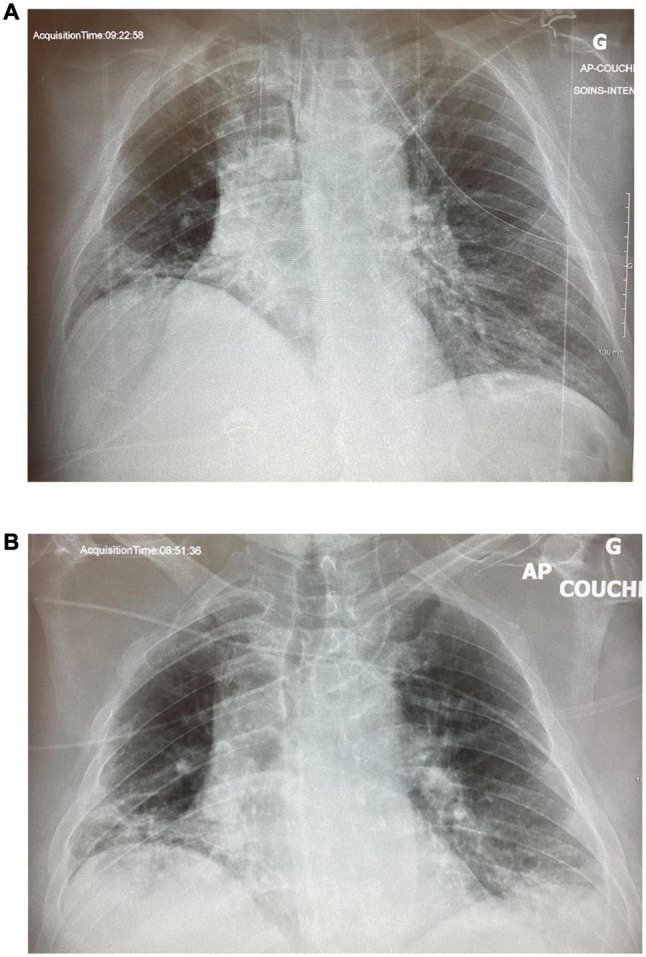
Case 3: **(A)** ICU Day 11. **(B)** CXR on day 14, 24 h post-extubation.

A 58-year-old man with obesity, hypertension, and dyslipidemia presented with a 1 week history of dyspnea and presented to a community hospital emergency room where he was put on high flow nasal cannulae and then transferred to our center for further management. On arrival, he was saturating 88% on 70% FiO_2_, and his initial CXR showed extensive bilateral infiltrates and volume loss (Figure 7A). He developed agitation, and his saturation deteriorated to the low 80’s despite increasing his FiO_2_ to 100%. He was intubated and put on CMV with a tidal volume of 500 ml, respiratory rate of 26, and a PEEP of 16 cm H_2_O with a resulting plateau pressure of 27 cm H_2_O and a driving pressure of 11 cm H_2_0. Paralytics were not required. He required a norepinephrine infusion to maintain sufficient perfusion pressures. The FiO_2_ was gradually reduced to 50%, but on day 2, his CXR showed no significant improvement (Figure 7B), and a transesophageal echocardiogram ruled out significant ventricular dysfunction and valvular disease. The decision was made to put him on APRV-TCAV™ for recruitment purposes, and his initial settings were 29/1.5/0/0.4. An expiratory hold on these settings showed a driving pressure of 10. On the next day, his CXR showed significant recruitment (Figure 8A), and on day 4, his FiO_2_ was down to 35% on settings of 29/1.5/0/0.4. An ABG showed pH of 7.48, PCO_2_ of 51 mmHg, and PO_2_ of 80 mmHg for a P/F ratio of 228. At this point, his norepinephrine drip had been weaned off, and the patient appeared stable. Several consultants suggested weaning, as the patient no longer met ARDS criteria; however, dropping the P_High_ transiently at the bedside resulted in significantly decreased release volumes with associated desaturations and an increase in the EtCO_2_, suggesting derecruitment. Consequently, the treating team judged that alveolar stability had not been reached, and his settings were reverted back to their previous values. On day 10, he was successfully “dropped and stretched” to 18/10/0/0.4, which was essentially equivalent to a CPAP 18 cm H_2_O. He was then extubated to HFNC and initially did reasonably well but 4 h later, desaturated and required reintubation. He was placed back on APRV-TCAV™ with the setting of 28/2.5/0/0.4. On day 11, norepinephrine, which had been required post reintubation, was weaned off, and again his ventilatory settings were adjusted to 22/2.5/0/0.4, and FiO_2_ was decreased to 25%. The CXR appeared well recruited (Figure 8B), and he was extubated on day 13 after several hours on a CPAP of 12 CmH_2_O (Figure 9A) and subsequently remained stable (Figure 9B). This patient never required paralysis and subsequently did well and was eventually discharged.

This case illustrates several key elements of APRV-TCAV™. First, one can see how little recruitment happened in the first 24 h of CMV, whereas an impressive amount occurred in the first 24 h of TCAV™. Second, the time factor in alveolar stability was clear, as it took several hours before desaturation was noted, despite the patient having been on only 25% FiO_2_ prior to the failed first extubation. While gas exchange was excellent, alveolar stability had not truly been achieved yet, which again illustrates the phenomenon of “apparent cure” when well-recruited patients may no longer fit the definition of ARDS but quickly decompensate with the desolation of ventilators support and pressures.

### Time-Controlled Adaptive Ventilation in Clinical Practice Today

In general, different ventilatory modes are used in different phases or severity of illness. It is difficult to estimate the frequency of APRV-TCAV™ use in the critical care community, but in our experience, while APRV as a mode is relatively well known, the TCAV™ approach seems to be less so. However, recent studies and even meta-analyses have been published, indicating that, at least in some centers, experience with it is growing (Zhou et al., 2017; Ibarra-Estrada et al., 2021). Strategically, one may use CMV as a primary mode during acute illness and pressure support during weaning. Several modes or strategies may be used as rescues, such as inverse ratio ventilation, high-frequency oscillation, or APRV. APRV-TCAV™ can be used as a primary or as a rescue mode, but the authors would caution against inexperienced users attempting it without experienced supervision particularly in the most fragile rescue cases.

## Conclusion

In the second wave onward, we had several patients who had an uneventful course with APRV-TCAV™ for the vast majority of their ventilation and were successfully extubated, a few who required ECMO, and some who passed away of non-pulmonary COVID complications, such as intracerebral hemorrhage and arterial thrombosis. Our experience is heterogeneous and uncontrolled, not one from which outcome data can be inferred, although it was clear in several cases that APRV-TCAV™ was able to oxygenate and rescue patients where a traditional lung-protective strategy had failed. Challenges abound with implementing TCAV™, the most critical ones being education and team buy-in. In inexperienced hands and minds, APRV-TCAV™ certainly has more challenges than a traditional LTV/ARDSnet approach, but in the authors’ experience, APRV-TCAV offers substantial physiological advantages that are worth the investment to understand and implement. What is needed is well-designed comparative trials to see if the promising cases in our experience can translate into a survival benefit if APRV-TCAV™ is used as a primary mode.

## Ethics Statement

Written, informed consent was obtained from the participant/s or next of kin for the publication of any patient-related data or information.

## Author Contributions

PR was the clinician who managed and reported cases described. BD contributed extensive writing of the main text and review of the document. Both authors contributed to the article and approved the submitted version.

## Conflict of Interest

PR and BD teach a mechanical ventilation workshop on the mode discussed. PR receives financial payments from the workshop.

## Publisher’s Note

All claims expressed in this article are solely those of the authors and do not necessarily represent those of their affiliated organizations, or those of the publisher, the editors and the reviewers. Any product that may be evaluated in this article, or claim that may be made by its manufacturer, is not guaranteed or endorsed by the publisher.
